# Frail elderly patients’ experiences of information on medication. A qualitative study

**DOI:** 10.1186/1471-2318-12-46

**Published:** 2012-08-22

**Authors:** Sara Modig, Jimmie Kristensson, Margareta Troein, Annika Brorsson, Patrik Midlöv

**Affiliations:** 1 Department of Clinical Sciences in Malmö, Family Medicine, Skåne University Hospital, Lund University, SE 20502 Malmö, Sweden; 2 Department of Health Sciences, Lund University, PO Box 157, SE 22100, Lund, Sweden; 3 Tåbelund Primary Health Care Centre, Solv. 33, S-241 31 Eslöv, Sweden

**Keywords:** Elderly, Medication knowledge, Information, Confidence, Qualitative, Content analysis

## Abstract

**Background:**

Older patients generally have only poor knowledge about their medicines. Knowledge is important for good adherence and for participating in decisions about treatment. Patients are entitled to be informed on an individual and adequate level. The aim of the study was to explore frail elderly patients’ experiences of receiving information about their medications and their views on how the information should best be given.

**Methods:**

The study was qualitative in design and was carried out in 2011. Twelve frail elderly (aged 68–88) participants taking cardiovascular medications participated in semi-structured interviews covering issues related to receiving information about prescribed medicines. The interviews were recorded, transcribed and subjected to content analysis, in which the text was analysed in five steps, inspired by Graneheim and Lundman.

**Results:**

The results revealed that the experiences which the elderly participants had regarding the receiving of medical information fell into two main categories: “Comfortable with information” or “Insecure with information”. The elderly felt comfortable when they trusted their physician or their medication, when they received enough information from the prescriber or when they knew how to find out sufficient information by themselves. They felt insecure if they were anxious, if the availability of medical care was poor or if they did not receive enough information.

**Conclusions:**

Factors that frequently caused insecurity about information and anxiety were too short consultations, lack of availability of someone to answer questions or of the opportunity to contact the physician if adverse effects are suspected. These factors could easily be dealt with and there must be improvements in the clinics if the patients´ feelings of security are to be increased.

## Background

Frail older people often receive treatment that entails a daily intake of several medicines [1,2]. It is of utmost importance and also a legally binding regulation, that the patients receive the optimal amount of information on a suitable level that will allow them to manage their medication [3].

Inadequate information and less medical knowledge can lead to poorer adherence to prescribed medication [4]. Not taking one’s medicines in the prescribed way can lead to a reduced therapeutic effect or overdose-related problems. These in turn can result in further medication, unnecessary investigations or hospitalization. Elderly patients taking cardiovascular medication are more vulnerable to noncompliant behavior since they often require multiple and long-term therapy. Taking several medicines can give rise to difficulty in remembering the indications or possible adverse effects for each of the medicines and in deciding when to sound the alarm. These people are also more vulnerable because of diminished plasticity due to their illnesses and age.

Knowledge about medication is significantly related to adherence [4], but is also important for the patient to be able to participate in decisions about the treatment and to cope with a chronic disease [5]. The knowledge that patients, and especially older patients, have about their medicines is generally poor [6-11]. In a previous Swedish study (n = 41) [12], 71% of the sample were able to state the indication for at least 75% of their medications but only 6% knew anything about possible adverse reactions or risks. Thirty-nine percent agreed with the statement “My medicines are a mystery to me” (part of the Beliefs about Medicines Questionaire, assessing attitudes towards medications [13]). Little is known, however, about why the knowledge concerning prescribed medication is so poor among frail elderly people and what can be done to improve the situation, which is important, since these people often have multiple illnesses, take many medicines regularly and consume a large amount of healthcare. As a care-giver to be able to help the patient to manage the medication, it is necessary to explore the patients’ experiences and understanding of their medicines. A Swedish focus group study of elderly patients revealed feelings of distrust and the existence of many unanswered questions. The elderly did not know the indications of their medicines and had worries about possible adverse effects. However, they did not raise many concerns with the physician [14]. In another Swedish study interviews were performed with elderly heart failure patients (n = 22) thirty days after having received a prescription for medication from a hospital [15]. Although all the patients received verbal and written information regarding their medication, shortcomings concerning the names of the medicines, doses and when the medicine was to be taken, were common. Although efforts were made to provide adequate information, sufficient knowledge was still not instilled. More knowledge is needed about patients´ experiences of receiving information about medicines, if care quality is to be improved and the possibilities for older people to take an active part in their treatment are to be increased.

The aim of this study was to explore elderly patients’ experiences of receiving information about their medications and their views on how information optimally is given.

## Methods

### Participants

The interviews were performed as part of a larger project, designed to evaluate the use of Case Manager as a care model for the elderly with multiple illnesses [16]. This was a randomized controlled study, which took place in a town in southern Sweden with 30 000 inhabitants, including both rural and urban areas. Those included were aged 65 and above, needed help with at least two activities of daily living, such as cooking, washing or personal hygiene, had been admitted to hospital at least twice, or had at least four contacts in outpatient or primary care, during the last twelve months. They were able to communicate verbally and had no cognitive impairments. An additional criterion for this study was receiving treatment with one or more cardiovascular medications, including Warfarin. Twelve persons were strategically selected from the main project as they were informative and talkative. The participants came from both groups in the main study (intervention and control), but were, in aspects concerning information of medication, judged not to be influenced by the intervention. The characteristics of the participants are presented in Table 1.

**Table 1:** Characteristics of the participants

**Participant no**	**Age**	**Gender**	**Number of medications**	**Marital status**	**Educational level**
P1	82	female	4	widow	compulsory education^1^
P2	76	female	7	divorced	vocational education^2^
P3	76	male	13	married	compulsory education
P4	81	female	11	widow	vocational education
P5	84	male	5	married	vocational education
P6	78	male	6	cohabitant	compulsory education
P7	72	female	3	married	vocational education
P8	87	male	10	widower	vocational education
P9	82	female	19	other	vocational education
P10	68	female	16	cohabitant	vocational education
P11	88	male	10	widower	vocational education
P12	80	female	4	widow	high school diploma

^1^ For subjects at this age school was compulsory for six years, from the age of seven to thirteen.

^2^ Vocational education comprised courses directed towards future employment in such fields as plumbing, tailoring, secretarial work etc. It did not involve taking an examination that would qualify the student for academic studies at the further education level.

### Procedure

The interviews took place between February and August 2011. A letter with information about the study and the interviews was sent to those selected. One week later the first author telephoned, gave additional information and asked if the person was willing to participate. All those invited agreed to do so. Five men and seven women were interviewed, aged 68 to 88 years. The semi-structured interviews were carried out in the participants’ homes by the first author, a GP who had rich experience of consulting but no previous experience of conducting qualitative interviews. She was not involved in the medical care of the participants. The interviews followed a thematic interview-guide, which comprised questions concerning medical treatment in general and specific questions about how they experienced receiving information about their prescribed medicines (Appendix A). The interview started with the question “Which cardiovascular drugs do you take?” and was then followed by more-in-depth questions. The duration of the interviews ranged from 23 to 55 min. During four of the interviews, the participant´s husband/wife was present and provided occasional comments. The interviews were tape-recorded and transcribed verbatim by a professional transcriptionist.

### Analysis

A qualitative content analysis was performed. According to Berg, content analysis may cover latent and manifest levels and a combination of the two. The manifest level concerns the surface of the text focusing on the more visible and obvious parts. The latent level comprises an interpretation in which deeper aspects of meaning are sought in the text [17]. The text was analysed in five steps, inspired by Graneheim and Lundman [18]. In the first step the interviews were read through and listened to several times by SM to gain a sense of the whole and to become familiar with the individual interviews. The other authors also read a couple of interviews in order to get a picture of the material. In the second step meaning units related to the aim were identified. In the third step the meaning units were condensed and labeled and finally coded on the basis of their content. This was done by SM and confirmed by JK and AB. Based on the codes, sub-categories and categories were developed in the fourth step. There was an ongoing dialogue between the authors throughout these steps and in the fifth step the categories were carefully discussed until two main categories could be identified (Table 2).

**Table 2:** Example of the analytical process

**Meaning unit**	**Code**	**Subcategory**	**Category**	**Main category**
I trust the doctor, that these are the right medicines for me	Confidence in the doctor, despite lack of information about side effects	Confidence in the medications and in the physician	Trust/confidence	Comfortable with information
The ordination you get from a doctor, I think you should stick to it and if it doesn´t help, you should call the doctor to get the green light. You shouldn´t medicate yourself.	You should adhere to the doctor´s ordination and not change it yourself	Compliance		
I am very satisfied, since I don´t miss any information, about anything, what kind of pill it is or why I get it and if she changes the dose she tells me why.	Very satisfied with the complete information from the doctor. Receives information when changes are performed and why.	Satisfactory information	Satisfaction with information	
.such as when I am called for an appointment with my doctor, then he isn´t at the ward but in the consulting room where we sit in peace and quiet and talk.	Information in peace and quiet during the next visit is appreciated	Timing of information		
-Have you got any information about possible adverse effects of these medicines?	*No information about possible adverse effects. Had to read about it herself.	Reading package leaflets	Taking control	
-No, I have read about it on the leaflets. And I have read there what they are good for.	*Read about indications on the leaflet			

After 12 interviews, there was consensus that saturation had been reached as no new categories reflecting the study aim could be developed from the data. The last two interviews were analysed without producing any additional change in the structure.

### Ethics

The Regional Ethical Review Board, Lund approved the project (no 342/2006; no 499/2008). Informed consent was obtained from all participants. The researcher conducting the interviews (SM) was not involved in the medical care of the patients.

## Results

**Figure 1:**
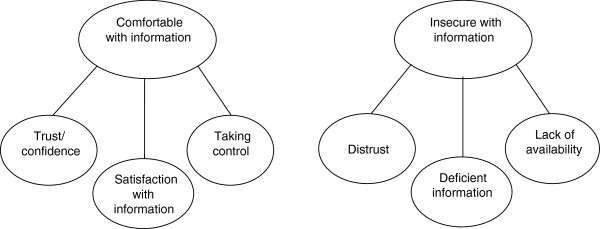
Main categories and categories.

The experiences concerning medical information were shown to fall into two main categories: “Comfortable with information” and “Insecure with information”. These were based on stepwise developed codes, sub-categories and categories, as described in the method section. The first main category implied having trust in the physician or the medication, receiving satisfactory information or having enough control to find out sufficient information alone. Experiencing insecurity implied feelings of distrust, insufficient information or lack of availability of a physician (Figure 1).

### Comfortable with information

The first main category “Comfortable with information” covered three categories: “Trust and confidence”, “Satisfaction with information” and “Taking control”.

### *Trust/confidence*

The text revealed feelings of security concerning the prescribed medicines. This was obvious when the participants felt confident about the information and trusted the care-giver. Experiencing that the medicines had the desired effect enhanced the trust. As one participant stated:

Of course you feel safe when you feel that this (the medicine) helps. (P12)

When confidence in the physician was high, the person had no wish for more information, although complete information was not always given for example about possible adverse effects. Feelings of trust and confidence were also seen when medicines were replaced after collaboration between physician and patient when adverse reactions occurred.

Trusting the physician also entailed adherence to ordination and not knowingly ignoring tablets. Changes in dosage were not performed by the patient in isolation. Cardiovascular medications appeared extra important. Medicines were often taken without question because of the obvious need, as exemplified by the following:

I have taken them quietly. It wouldn´t work otherwise. It is evident as soon as I don´t take them. (P12)

When the participants felt secure, they were unafraid of generic exchanges of drugs. Instead, as they knew that the content was the same, the cheaper price was regarded as positive. The pharmaceutical personnel wrote on the packaging explaining what had been exchanged. The elderly trusted the pharmacy if the information was satisfactory and the different names were manageable if the explanation was clear.

When confident, the participants experienced having no special treatment or less information due to high age, except for people with dementia. Opinions were expressed that older people were sometimes placed second when it came to operations, but not regarding information.

### *Satisfaction with information*

When information about the medicines was given in a satisfactory way, the participants felt comfortable. It was shown to be important to get detailed answers to questions. The answers should be given at the appropriate level and without using difficult medical terms. To be satisfied also meant receiving information about changes in prescription or doses and why the medication was changed. The partner could be included when information was given and this was seen as providing extra security. A participant could be aware that sometimes the information was limited but despite this they were satisfied and did not ask for further details. It was considered a bonus if the doctor made an extra call after any changes to see how the patient felt and if any adverse effects had occurred, as cited:

This physician has called since he changed these pills several times of course. And then he asked " How are you today? Can you tolerate it?" It was wonderful to be treated that way. (P4)

It was a common experience that the physician checked if there were any questions or possible adverse effects at each annual review of the prescription, which enhanced satisfaction. However, it was also stated that information did not necessarily have to follow each prescription, but only when a new medicine was prescribed. Some participants even thought that information was not necessary at all when prescriptions were renewed and that if questions arose, one could always call.

The timing of information was important for the experience of satisfaction. It was not always seen as good to receive new information during hospitalization, for example. Information about new prescription should be given on discharge or during an early revisit and preferably in peace and quiet. On the other hand, wishes were also expressed for more information about the indication for and adverse effects of new medicines from the hospital physician before the nurses dispensed them.

. .then I would of course like to be informed, maybe not at the hospital - then you are so excited and scared you cannot take in any information at all. But at the first revisit perhaps. So you would get appointments fairly quickly to the doctor who has prescribed medications or the treating physician that you are assigned, to have the opportunity to discuss drugs. Which is good and which is bad and also what side effects might occur. (P7)

Information about indication was usually given when a new medicine was prescribed. The participants were satisfied when they received explanations about how the medicines worked but also when they received more limited information - what disease the medicines were for, but not how they worked. The participants preferred the physician to explain how he/she thought and why the medicine was prescribed.

When I get a new pill, she usually goes through it with me; she usually says what it is good for and how it works and such things. And I should watch if I have something more than what is written in the leaflet. If something else happens. (P8)

### *Taking control*

When more information was needed, the participants could still feel comfortable if they had strategies for finding the necessary information. This way of taking control was seen for example when the participants used the leaflet that came with the package as a complementary source of information and read it thoroughly; especially when a new medicine was prescribed. Information about both indication and possible adverse reactions was noted. It was considered safer to have this information in advance in case side effects should occur. The participants who began to experience forgetfulness, wanted to reinforce the information given by reading the package leaflet, as exemplified here:

I´m so forgetful of course. So maybe it｀s best for me to read and repeat it, isn´t it? Yes, it is, since I would have forgotten what they said. (P3)

Another strategy for finding information was to use the pharmacy. Sometimes the information available there was insufficient, but often the participants reported satisfaction with the information given by the pharmacy and thought that it was just enough. The pharmacy personnel were aware of when there was a need for further information. They concentrated on new medicines and always asked if the patient knew how it worked, as exemplified here:

They do of course recognize that you've been there so often and got that medicine, so they usually ask if you have taken it before, if you know how it works and so on. If you say yes, they have no reason to say anything. But if it is a new medicine they might say: " Now remember that you should take it with meals and so on”. (P7)

The text showed that several sources of information were used to take control and increase knowledge and security, for example magazines, medical programs on TV or (more seldom) the Internet. Some read the Swedish Medicines Compendium for Physicians (FASS), and others asked relatives. One important source of information was the medicine container. It was often possible to read both the indications for therapy and the dosage from the label.

The most usual way to compensate for forgetfulness was to use a medication organizer, filled weekly. Many also used a medicine list to keep track of medications.

It was commonly stated that it was the patient´s responsibility to ask questions about side effects for example and also to question the therapy more often. This was one way of exercising control. Some participants made extensive investigations about new medicines before taking them. On the other hand, the vast majority argued that it was the doctor´s duty to supply them with information. Below is an example of questioning:

So therefore, I would like to ask when he comes the next time " Do I have to take all these?” (P6)

### Insecure with information

The second main category “Insecure with information” covered three categories: “Distrust”, “Deficient information” and “Lack of availability”.

### *Distrust*

Feelings of insecurity about the information were closely linked with feelings of distrust towards the health system. It was shown, for example, when participants had thoughts about their medicines but did not question the physician. It also included having concerns about the amount of drugs and if all the drugs were necessary. In general the participants had great confidence in the heart drugs and Warfarin, but were often skeptical about their other drugs and thought that maybe prescription renewal was just a routine. According to one participant:

Safe…I don´t feel completely safe since I would like to know if I can remove any of them. (P6)

Distrust could also be directed towards drugs in general. The participants wanted to have enough knowledge about their medicines*,* but did not want to be afraid of the medicines because of having too much information. They believed that you could easily imagine the symptoms/side effects if you read too much, as exemplified here:

I happened to read that and I think, you know, when you read those then you have almost everything. (P2)

Although most of the participants did not feel they were discriminated against because of their age, the opposite was also experienced: that the doctor paid less attention because they were old. There were feelings that the healthcare sector did not care about old people, that the elderly were given lower priority and that hardly any time was available for education and information for the elderly, which all contributed to feelings of distrust.

Distrust could also mean being afraid of not receiving the right treatment due to a generic prescription. There was an uncertainty about getting it right when the tablets looked different each time. Getting tablets that “did not have the right name” caused feelings of insecurity. The new names of the tablets made it difficult to remember the indication for the treatment. The participants saw the risks of mixing up the medications, especially when they were tired or cognitively impaired, of taking the wrong thing or too much. Some participants preferred to pay a little more and get the same drug every time. There was a fear of new additives and of inferior quality if one had cheaper replacement drugs.

You are so used to having one sort, you know, and then you get another the next time you come. It may not be the same as you had before. Then you become a little confused - what is the order now? (P9)

### *Deficient information*

The information about medicines was sometimes deficient and this caused insecurity about medication use. The physicians did not always explain in detail, or even at all, why a new medicine was prescribed or how it worked. The participant often received a short piece of information, such as “for the heart”, but no more extensive explanation. Many also reported that they had never received any information from the doctor about the possible side effects of their medications. They had to find out themselves or rely on the package leaflet. Questions asking for more details about indication or side effects could even be ignored.

. .and often if you ask about side effects, so yes, “there are side effects for all medications and there are no more from these than from the other and there is so much in FASS but it´s not certain that everyone has it”. That's basically the answer you have gotten when you have asked. (P7)

It was commonly experienced that the information about new medicines given in the hospital was too brief. The patients often received their medicines from the nurses without any information from the doctor about why a new medicine or dose was introduced. At the end of a hospital stay, there was not always a discharge talk with the responsible doctor, even though the medicine list was changed. Instead a note was given by the nurse.

.you know at the hospital, they are in a hurry, they come in with their lists, and then it´s thanks and goodbye. (P4)

Many of the participants expressed a wish for more or better information. They wanted someone to prioritize the data, tell them about the indication, provide an opportunity for contact if there were suspected adverse reactions or an offer of an appointment just to discuss the medicines. If the information was limited and there was no one available to answer questions, there were soon feelings of insecurity. Wishes were also expressed for more information about why a medicine was sometimes withdrawn.

### *Lack of availability*

If access to the physician´s help was unsatisfactory, feelings of insecurity emerged. Lack of time was often a major obstacle to the doctor giving information. There was not time enough to ask the doctor all their questions. The text revealed strong feelings of disappointment – only what was most important or acute was prioritized during an appointment. There was barely time for anything other than a specific problem and certainly not for discussing medicines. It was difficult to ask about possible side effects, since the physician was always in a hurry when giving information. The yearly check-up mostly comprised the physical examination, with no possibility to ask questions. However, the physician did say if anything about the medicines was to be changed. The text also contained reports of the lack of doctor´s time in the hospital. For this reason the patients were often given new medicines by the nurses without first receiving information from the physician, such as why it was given and if there were any possible side effects.

I know that doctors are so short of time you don´t have time for anything except the most necessary. And, it's a bit dull. (P10)

Many participants asked for continuity in the doctor´s contact and thought that lack of physician continuity could affect the giving of medical information. One participant stressed that new physicians only focus on the acute problem:

You are not likely to meet the same doctor when you go to the clinic. And usually it's something new, acute that causes the visit. Then there is time only for the acute part. (P10)

The text revealed that it was sometimes difficult to reach the doctor at the time the problems arose. The participants did not want to wait for their doctor. It was also clear that prescriptions were often renewed without any further information or contact with the doctor. This was not a problem for many of the participants – they asked for information only if anything was unclear – but some participants wanted the doctors to check if there were any questions or problems.

## Discussion

This qualitative study on how frail elderly people experience receiving information about their medications shows that two main categories might describe this situation: being comfortable with the information given and feeling secure and being insecure with information. All interviewees contained feelings of security as well as insecurity, but every category or sub-category was not present in every interview.

Optimal information is meant to create more knowledge among patients and as Keohane et al. express it: “Knowledge confers a sense of control, and enhances the ability to cope with chronic disease. Knowledge is power, whereas fear of the unknown is detrimental, and is likely to lead to poor adherence to prescribed management regimes [5].” The feelings of security in the first main category in this study were related to optimal information, but also to this sense of control; the participants had the tools that allowed them to find the information about their medicines if that given by the physician was insufficient. The package leaflets were the natural place to search information, but the pharmacy and mnemonic lists were also used. However, in an optimal situation, the physician should be the primary source of information, as is also stated by Gordon et al. [19].

The patients also felt secure if they had complete confidence in the physician or the medications. It was obvious that not all patients tried to obtain full knowledge. They were, perhaps, too frail to manage to go into the details and that is not necessarily wrong. As a prescriber, it is important to remember that there is no “ideal” patient. Some people, especially if they are older, might find it frightening to be involved in decision-making and to be supposed to have enough knowledge to do so. However, a level of knowledge admitting the ability to differentiate between the symptoms of illness and adverse effects of the medication is valuable to all patients. Adherence is supposed to be good if there is trust in the physician or the medication. According to a Norwegian study, only 17% of elderly patients receiving home nursing wanted to know more about their medications, but 75% showed high or medium adherence, perhaps indicating confidence in the care [20]. A study from New Zealand reported mainly positive beliefs about medicines among older people in their own homes, although many had worries about adverse effects, probably due to high confidence in the care and in the medications even here [21].

This high level of confidence may also indicate that the present generation of elderly people often does not take an active part in decision-making. They do not question their therapy; they listen to those in positions of authority and think that the doctor knows what the best is for them. A study in a nursing home setting in Ireland revealed that all residents (n = 17) accepted control of their medication without question and did not appear to want involvement in prescribing decisions or the administration of their own medicines [22]. In the future patients will probably be more demanding and also more questioning. For an optimal and rewarding discussion between the physician and the patient and a positive involvement in medical decisions, information will be even more important. However, according to Swedish law, the current patients are already entitled to be involved and adequately informed.

The results of our study are in line with the finding of another recent Swedish study, showing that the participation of the elderly in their medical care is primarily a question of good communication and information. It is essential that enough time is allowed for information [23]. Our participants pointed out the importance of having enough time to ask the doctor everything they wondered about. They wanted opportunities to discuss the lingering medications and not just short visits focusing on acute problems. The lack of access to a doctor caused insecurity. This is also shown in a study by Moen et al. [14], where the respondents expressed disappointment with consultations that were too short and allowed no time for questions. The timing of information was also important: informative revisits after hospital stays were preferred to information given when they were upset due to a new diagnosis or an acute condition. This is in accordance with the interviews with heart-failure patients in the UK [24] as well as the findings of Gordon et al. [19]. Repeated information was also appreciated, since forgetfulness was common. The prescriber should remember this – initial information alone is not enough; it should be repeated when prescriptions are renewed. Several of the respondents were treated with Warfarin and it can be assumed that extensive information was given when the therapy was initiated, since this is done according to regulation in Sweden. However, it was commonly reported in the interviews that no information about possible adverse effects was given. The initial information had obviously not been absorbed by the patients and should therefore be repeated. This was also stated in a study of an atrial fibrillation population treated with Warfarin, were there was a general knowledge deficit regarding Warfarin therapy [25]. Educational efforts must be made frequently to avoid the negative impact of Warfarin effect, which can be severe.

The second main category, insecure with information, included feelings of distrust and disappointment due to insufficient information. Information about indications for the treatment was lacking in some cases. The information on the medicine packages was sometimes too brief - “for the heart” – and gave no details, explaining why the drug was prescribed. This was also reported by Moen et al. [14] and Gordon et al. [19].

Information regarding possible side effects was often deficient, in accordance with the findings of several other studies [14,19,26]. It was commonly reported that the issue of side effects was never discussed – not only at the time the prescription was written but also even when the patients tried to ask about it. Perhaps the physicians' fear that the patients would not dare to take the medicines if they were aware of possible side effects. Some respondents also admitted that one could easily imagine symptoms if one read about possible side effects. Gordon also reported that while side effects were a source of concern for many people, only a few brought the issue up for discussion [19]. This might be due to the feelings that the issue is often neglected.

The findings in this study, that information about new medicines prescribed by the hospital is deficient have been previously found. According to Micheli et al., knowledge about new medicines prescribed in hospital was significantly lower among patients who had been treated at the hospital for a long time [27]. Many of our participants reported that changes were made and new treatments were introduced without any information being given by the responsible physician. In the USA 90% (n = 89) of internal medicine residents noted that they had never been told of any adverse effects of new medicines initiated during hospitalization [28]. This may be due to lack of time, which was also suggested by some of the respondents in our study. But since medical treatment is an important part of the care and good adherence is needed for a therapeutic effect to be achieved, time for adequate information must be prioritized. Micheli also reported that knowledge was lower among the elderly (<80). Do physicians pay less attention to the elderly and spend less time giving them information? Our study revealed feelings of discrimination on grounds of age in some cases, although the majority of the participants had no such feelings.

Situations caused by patients, that most often lead to side effects and secondary hospitalization occur in the areas of administration of drugs, dosage modifications or failure to follow clinical advice [29]. One may conjecture that this is due to insufficient knowledge. The problem of deficient information at the time of prescription must be acknowledged and the information situation must be improved, both at hospitals and at the health centers. Colledge et al. suggest the use of strategies such as “The teach back method”, where patients are given information and are then asked to rephrase for the physician, offering the chance to correct mistakes and check comprehension. They also propose guiding the patient to reliable sources of information [30].

This study has its strengths and weaknesses. The design is qualitative which implies that it should be assessed by means of trustworthiness [31], which comprises credibility, transferability, conformability and dependability. Credibility refers to the truth and the believability of the data and on whether the results are based on faithful descriptions. The conditions for data collections, interviews, sampling and how well the data are covered in the main categories and categories are important aspects to consider [18]. All the authors participated in the analysis process and this triangulation increases credibility. In addition the analytical process is made transparent in Table 2 and by the use of quotations from the interviews in the results. The interviews were performed in the participants´ homes which may be supposed to strengthen the method since the home environment should be seen a safe place in which to talk freely. Some interviews were rather short, lasting approximately 25 min, which might have influenced their depth. However, the mean length of the interviews was 37 min, and the content of the texts was judged to be detailed and rich. Efforts were made to create good conditions for the interviews. Both men and women with varying amounts of medication contributed to sample variation (Table 1). Thus, the results can be viewed as credible. Data were collected using a semi-structured interview guide. It has been suggested that this strengthens trustworthiness [18]. The guide ensured that the participants were asked questions about the same areas. The method used, makes it impossible to generalize our findings to other populations but Lincoln and Guba have suggested that in such studies the reader must judge transferability; whether the findings are useful in a specific population. We have elucidated the experiences of frail elderly patients.

More research in this area is needed and the results must be communicated to physicians. Identifying the types of misunderstandings about medications that can lead to side effects or a poorer therapy outcome in the elderly, will make it easier for physicians to direct interventions and optimize information.

### Clinical implications

Since patients who feel secure and comfortable with both information and their medications are considered to show greater adherence, it is desirable that such feelings should be engendered. Patients are also entitled to be informed at an individually optimal level. Adherence is particularly important for the elderly, as they often suffer from chronic diseases requiring long-term therapy. Factors that often cause insecurity about information and anxiety were too short consultations, discontinuity, lack of availability for questions or opportunity to contact the physician if adverse effects were suspected. These are factors that could easily be dealt with and there must be improvements in the clinics if the patients´ feelings of security are to be increased.

## Conclusions

The results of this study reveal two main categories of medical information in elderly participants: “Comfortable with information” or “Insecure with information”. The elderly felt comfortable when they trusted their physician or their medication, when they received enough information from the prescriber or when they had the tools to find out enough information by themselves. They felt insecure if they were anxious, if the availability of medical care was poor or if they did not get enough information.

## Appendix A

### Semi-structured interview-guide

Which medications do you take for heart disease?

What do you know about their effects?

What have you been told about possible adverse effects?

Which medicine do you think is the most important for you?

What have you been told about these medications? By whom? When? Tell me about…

What information did you get the first time the medicine was prescribed?

Are you satisfied with the information you have received? Why? Why not?

Would you have preferred to be informed in another way? Or more extensive?

How would you like to be informed?

What kind of information is usually given to you by your doctor when the prescriptions are renewed?

Do you go yourself to the pharmacy to buy your medicine?

Do you read the package leaflet? Do you look for information about your medicines elsewhere?

Do you use a medicine organizer? If yes, who is preparing it – you, a relative, a district nurse? Or do you take the tablets directly from the package?

Many people sometimes skip a tablet. Does it happen to you? How come? Then how do you reason?

How do you feel about, at the pharmacy, being given medications which do not have the same names as the ones you got information about?

## Competing interests

The authors declare that there are no competing interests.

## Authors' contributions

SM participated in the design of the study, carried out the interviews, performed the analysis together with JK and all the other authors and drafted the manuscript. JK contributed significantly to the analysis and the revision of the manuscript. MT and AB contributed strongly to the design of the study and participated in the analysis. PM participated in the design of the study and in the analysis. All authors read and approved the final manuscript.

## Pre-publication history

The pre-publication history for this paper can be accessed here:

http://www.biomedcentral.com/1471-2318/12/46/prepub
